# The induction of synaesthesia with chemical agents: a systematic review

**DOI:** 10.3389/fpsyg.2013.00753

**Published:** 2013-10-17

**Authors:** David P. Luke, Devin B. Terhune

**Affiliations:** ^1^Department of Counselling & Psychology, University of GreenwichEltham, UK; ^2^Department of Experimental Psychology, University of OxfordOxford, UK

**Keywords:** drugs, serotonin, synaesthesia

## Abstract

Despite the general consensus that synaesthesia emerges at an early developmental stage and is only rarely acquired during adulthood, the transient induction of synaesthesia with chemical agents has been frequently reported in research on different psychoactive substances. Nevertheless, these effects remain poorly understood and have not been systematically incorporated. Here we review the known published studies in which chemical agents were observed to elicit synaesthesia. Across studies there is consistent evidence that serotonin agonists elicit transient experiences of synaesthesia. Despite convergent results across studies, studies investigating the induction of synaesthesia with chemical agents have numerous methodological limitations and little experimental research has been conducted. Cumulatively, these studies implicate the serotonergic system in synaesthesia and have implications for the neurochemical mechanisms underlying this phenomenon but methodological limitations in this research area preclude making firm conclusions regarding whether chemical agents can induce genuine synaesthesia.

## Introduction

Synaesthesia is an unusual condition in which a stimulus will consistently and involuntarily produce a second concurrent experience (Ward, 2013). An example includes grapheme-color synaesthesia, in which letters and numerals will involuntarily elicit experiences of color. There is emerging evidence that synaesthesia has a genetic basis (Brang and Ramachandran, 2011), but that the specific associations that an individual experiences are in part shaped by the environment (e.g., Witthoft and Winawer, 2013). Further research suggests that synaesthesia emerges at an early developmental stage, but there are isolated cases of adult-onset synaesthesia (Ro et al., 2007) and it remains unclear whether genuine synaesthesia can be induced in non-synaesthetes (Terhune et al., 2014).

Despite the consensus regarding the developmental origins of synaesthesia, the transient induction of synaesthesia with chemical agents has been known about since the beginning of scientific research on psychedelic drugs (e.g., Ellis, 1898). Since this time, numerous observations attest to a wide range of psychoactive substances that give rise to a range of synaesthesias, however, there has been scant systematic quantitative research conducted to explore this phenomenon, leaving somewhat of a lacuna in our understanding of the neurochemical factors involved and whether such phenomena constitute genuine synaesthesia. A number of recent theories of synaesthesia implicate particular neurochemicals and thus the possible pharmacological induction of synaesthesia may lend insights into the neurochemical basis of this condition. For instance, *disinhibition* theories, which propose that synaesthesia arises from a disruption in inhibitory activity, implicate attenuated γ-aminobutyric acid (GABA) in synaesthesia (Hubbard et al., 2011), whereas Brang and Ramachandran (2008) have specifically hypothesized a role for serotonin in synaesthesia. Furthermore, the chemical induction of synaesthesia may permit investigating experimental questions that have hitherto been impossible with congenital synaesthetes (see Terhune et al., 2014).

Despite the potential value in elucidating the induction of synaesthesia with chemical agents, there is a relative paucity of research on this topic and a systematic review of the literature is wanting. There is also an unfortunate tendency in the cognitive neuroscience literature to overstate or understate the possible induction of synaesthesia with chemical agents. The present review seeks to fill the gap in this research domain by summarizing research studies investigating the induction of synaesthesia with chemical agents. Specifically, our review suggests that psychoactive substances, in particular those targeting the serotonin system, may provide a valuable method for studying synaesthesia under laboratory conditions, but that methodological limitations in this research domain warrant that we interpret the chemical induction of synaesthesia with caution.

## Methods

### Literature search and inclusion criteria

A literature search in the English language was conducted using relevant databases (PubMed, PsychNet, Psychinfo) using the search terms synaesthesia, synesthesia, drug, psychedelic, LSD, psilocybin, mescaline, MDMA, ketamine, and cannabis and by following upstream the cascade of references found in those articles. Initially a meta-analysis of quantitative findings was planned, however, it became apparent that there had been only four *direct* experimental attempts to induce synaesthesia in the laboratory using psychoactive substances, making such an analysis unnecessary. A larger number of other papers exist, however, describing *indirect* experiments in which participants were administered a psychoactive substance under controlled conditions and asked via questionnaire, as part of a battery of phenomenological questions, if they experienced synaesthesia during the active period of the drug. Whilst these studies typically provide a non-drug state condition for comparison they did not set out to induce synaesthesia and so are less evidential than direct experimental studies. There also exist a number of case reports describing the induction of synaesthesia using chemical agents within various fields of study. Under this category, we include formal case studies as well as anecdotal observations. A final group of studies used survey methodologies, providing information regarding the prevalence and type of chemically-induced synaesthesias among substance users outside of the laboratory. Given the range of methodologies and quality of research, we summarize the studies within the context of different designs.

### Drug types

The majority of the studies and case reports relate to just three psychedelic substances—lysergic acid diethylamide (LSD), mescaline, and psilocybin. However, some data is also available for ketamine, ayahuasca, MDMA, as well as less common substances such as 4-HO-MET, ibogaine, *Ipomoea purpurea*, amyl nitrate, *Salvia divinorum*, in addition to the occasional reference to more commonly used drugs such as alcohol, caffeine, tobacco, cannabis, fluoxetine, and buproprion.

## Results

The final search identified 35 studies, which are summarized in Table 1. Here we review the most salient results from the different studies.

**Table 1:** **Summary of studies reporting induction or modulation of synaesthesia through drug use**.

**Author(s) (year)**	***N***	**Study design**	**Stimuli**	**Drug(s)**	**Type(s) of synaesthesia**	**Prevalence of synaesthesia and other results**
Kelly (1934)	5	Experiment (direct)	Pure sonic tones (previously paired to colors)	Mescaline	Haptic-visual, kinaesthestic-visual, algesic-color	80%; No effect of tone-color training
Hartman and Hollister (1963)^a^ Hollister and Hartman (1962)	18	Experiment (direct)	Pure sonic tones	Mescaline	Auditory-visual	10%; Mescaline > no drug
Hartman and Hollister (1963)^a^ Hollister and Hartman (1962)	18	Experiment (direct)	Pure sonic tones	LSD	Auditory-visual	15%; LSD > no drug
Hartman and Hollister (1963)^a^ Hollister and Hartman (1962)	18	Experiment (direct)	Pure sonic tones	Psilocybin	Auditory-visual	11%
Masters and Houston (1966)		Experiment (direct)	Objects	LSD	Music-visual, color-gustatory, color-auditory, auditory-gustatory, music-olfactory	
Simpson and McKellar (1955)	4	Experiment (direct)	Various	Mescaline	Auditory-visual, kinaesthetic-visual, tactile-visual, olfactory-visual, algesic-visual, thermal-visual, olfactory-tactile, visual-thermal	Enhancement of congenital synaesthesia
Addy (2010)	30	Experiment (indirect)	Ordinary environment	*Salvia divinorum*	Visual-kinaethetic/propioceptive/somatic/tactile	57% (all types), 23% (visual>tactile)
de Rios and Janiger (2003)	930	Experiment (indirect)		LSD	Auditory-visual, auditory-color-tactile-kinaesthetic-emotion-concept	
Carhart-Harris et al. (2011)	9	Experiment (indirect)	Ordinary environment	Psilocybin	Auditory-visual	
Lahti et al. (2001)	18	Experiment (indirect)		Ketamine		
Riba et al. (2004)	18	Experiment (indirect)		Ayahuasca	Auditory-visual	28%
Savage et al. (1969)	22	Experiment (indirect)		*Ipomoea purpurea*		
Studerus et al. (2010)	327	Experiment (indirect)		Psilocybin	Auditory-visual	37%
Studerus et al. (2010)	102	Experiment (indirect)		MDMA	Auditory-visual	10%
Studerus et al. (2010)	162	Experiment (indirect)		Ketamine	Auditory-visual	27%
Studerus et al. (2011)	110	Experiment (indirect)		Psilocybin	Auditory-visual	Linear, dose-dependent induced synaesthesia
Studerus et al. (2012)	261	Experiment (indirect)		Psilocybin	Auditory-visual	Induced synaesthesia predicted by drug dose, absorption (Tellegen and Atkinson, 1974), alcohol consumption, sociability, emotional excitability, and activity
Brang and Ramachandran (2008)	2	Case report		Fluoxetine		Inhibition of congenital synaesthesia
Brang and Ramachandran (2008)	1	Case report		Buproprion		Inhibition of congenital synaesthesia
Brang and Ramachandran (2008)	1	Case report		Melatonin	Grapheme-color	Inducer-concurrent consistency in induced synaesthesia
Breslaw (1961)	1	Case report	Various	Psilocybin	Auditory-olfactory, color-auditory, gustatory-semantic	
Ahmadi et al. (2011)	1	Case report	Visual stimuli	Meth-amphetamine	Color-voices	Inhibited by electroconvulsive therapy
Cytowic (1993)	1	Case report		Amyl Nitrate	Gustatory-tactile	Enhancement of congenital synaesthesia
Cytowic (1993)	1	Case report		Alcohol	Gustatory-tactile	Enhancement of congenital synaesthesia
Cytowic (1993)	1	Case report		Amphetamine	Gustatory-tactile	Attenuation of congenital synaesthesia
Cytowic (1993)	1	Case report		Alcohol cessation		Inhibition of congenital synaesthesia
Fotiou (2012)	1	Case report	Music	Ayahuasca	Music-visual	
Klüver (1966)		Case reports		Mescaline	Auditory-visual, auditory-tactile, visual-tactile, color-gustatory, visual-auditory, concept-olfactory, auditory-somatic, visual-somatic, auditory-shape, auditory-tactile, visual-tactile, visual-thermal, haptic-visual, auditory-visual-somatic, auditory-visual-somatic-algesic, visual-tactile-conceptual-visual-gustatory-olfactory-entoptic	
La Barre (1975)		Case report		Mescaline	Visual-auditory	
McKellar (1957)	1	Case report		Mescaline	Auditory-gustatory	
Marks (1975)		Case reports		Cannabis and LSD	Auditory-visual	
McKenna (1982)	1	Case report		LSD	Auditory-shape	
Pahnke and Richards (1966)		Case reports		LSD	Music-color	
Popik et al. (1995)		Case reports		Ibogaine	Auditory, olfactory and gustatory	
Smythies (1953)		Case reports		Mescaline	Auditory-visual, auditory-emotion	
Hofmann (1983)	1	Case report		LSD	Auditory-visual	
Ward (2008)	1	Case reports		LSD	Visual-breathing	
Shanon (2003)		Case reports		Ayahuasca	Auditory-visual, olfactory-visual, tactile-visual	
Cytowic and Eagleman (2009)	1279	Survey		Alcohol		9% enhanced, 6% reduced
Cytowic and Eagleman (2009)	1279	Survey		Tobacco		1% enhanced, 1% reduced
Cytowic and Eagleman (2009)	1279	Survey		Caffeine		9% enhanced, 3% reduced
DeGracia (1995)	62	Survey		Multiple psychedelics	Auditory-visual, music-visual, Visual-auditory, visual-gustatory, auditory-somatic, color-olfactory, auditory-color, auditory-shape, auditory-touch, auditory-olfactory, auditory-gustatory, visual-music, color-gustatory, color-auditory, color-somat, gustatory-color, music-touch, music-somatic, music-shape, music-color, olfactory-gustatory, olfactory-visual	50% (all types)
Kjellgren and Soussan (2011)	25	Survey		4-HO-MET	Gustatory-auditory and auditory-visual	
Tart (1975)		Survey		Cannabis	Music-color	56% (all types)

Empty cells indicate that the respective information was not reported in the study.

a These two papers describe different analyses from the same data.

### Experimental studies

Among experimental studies, we distinguish between *direct* and *indirect* experiments as those that explicitly attempted to induce synaesthesia in a hypothesis-driven manner and those that explored the induction of synaesthesia as part of a larger battery, respectively.

#### Direct experiments

We identified four published experimental studies formally attempting to induce synaesthesia with pharmacological agents (Kelly, 1934; Simpson and McKellar, 1955; Hartman and Hollister, 1963; Masters and Houston, 1966). In the first study (Kelly, 1934), four non-synaesthetes had previously taken part in a 7-week auditory tone-color associative training study in which they were presented with eight different tone-color pairs 2000 times. Although the participants demonstrated a tone-color association learning effect, no evidence of spontaneous color photisms in response to the tones were observed. One week after the last training session the participants were joined by a fifth who had the day before received 1000 single tone-color pairings but likewise demonstrated no synaesthesia, and they consumed 15 g of (presumably dried) peyote cactus. Although not specified, this amount of peyote provides an estimated dosage of between 0.15 and 1.2 g of mescaline (Crosby and McLaughlin, 1973; Bruhn et al., 1978), providing anything from a mild to a very strong dose depending on relative alkaloid content (Shulgin and Shulgin, 1991). One participant took a further 5 g after receiving no visual perceptual changes, but to little effect. Although four of the five participants perceived colorful visual imagery, due to the mescaline, none perceived the appropriate color when the tones were played. However, these four participants experienced other types of synaesthesia, including haptic-visual, kinaesthetic-visual (especially color), and algesic-color. This suggests a high prevalence of synaesthesia following the consumption of mescaline, but that mescaline does not seem to enhance trained associations.

The second study (Simpson and McKellar, 1955), included two congenital synaesthetes (auditory-visual and multiple types) with the researchers acting as non-synaesthete controls (McKellar, 1957). Participants were administered four mescaline doses between 0.3 and 0.5 g (considered strong doses; Shulgin and Shulgin, 1991) on separate occasions. During the active effects of the drugs, participants were presented with a variety of sensory stimuli (visual, auditory, tactile, gustatory, olfactory, kinaesthetic, thermal, and algesic). Participants reported several distinct types of novel synaesthesias: auditory-visual (all 4 participants), kinaesthetic-visual (3), tactile-visual (2), olfactory-visual (2), algesic-visual (2), thermal-visual (1), olfactory-tactile (1), visual-tactile (1), and visual-thermal (1), with a relatively equal ratio of novel synaesthesias (10:7) between synaesthetes and non-synaesthetes. In addition, one of the congenital synaesthetes reported enhancement of their usual auditory-tactile and visual-thermal synaesthesias, suggesting that mescaline can both induce synaesthesia among non-synaesthetes and enhance synaesthesia among congenital synaesthetes.

A third study by Hartman and Hollister (1963; see also Hollister and Hartman, 1962) compared the effects of mescaline (5 mg/kg), psilocybin (150 mcg/kg), and LSD (1 mcg/kg), considered to be light to moderate doses, administered under blind conditions. A total of 18 participants took both substances 1 week apart and received auditory stimulation from 16 pure sonic tones before and after drug administration. Overall, participants reported significantly more colors and other auditorily-driven visual effects (brightening of the visual field, shattering of patterns, and patterning of form) compared to baseline whilst under the influence of both LSD and mescaline, but not psilocybin, although there was a non-significant increase in such experiences with the latter. Fewer than 50% of the participants (exact proportion not reported) experienced auditorily-induced synaesthesia under the influence of the three psychedelics.

A fourth experimental study is described in Masters and Houston (1966), but only minimal details about the methodology and results were presented. Specifically, they report a series of informal experiments and interviews conducted with 214 participants in 204 sessions in which psychedelic drugs were consumed. In the course of this work the authors report successfully intentionally inducing color-sound and auditory-gustatory synaesthesia with LSD, but there is no information regarding the proportion of participants reporting such effects, the dosage, or other results. Insofar as few details are provided about this study, it is difficult to critically evaluate its methodology and results, but the reported results do converge with the three previous experimental studies.

These four studies suggest that synaesthesia can be induced in a controlled environment using chemical agents. Nevertheless, they suffer from a large number of limitations including a lack of placebo control, double-blinds, and randomized allocation. The absence of these experimental controls warrant concerns about demand characteristics (Orne, 1962)—participants may have expected to have synaesthesia under the influence of the drugs, thereby inflating their tendency to endorse that they had experienced synaesthesia. In addition, the studies identified the experience of synaesthesia using self-reports rather than behavioral measures of automaticity or consistency (for a review, see Ward, 2013).

#### Indirect experiments

Despite the lack of direct experiments in the last 50 years, we identified nine studies that investigated the psychological effects of psychedelic drugs under controlled conditions, including placebo controls. As part of a broad assessment, these studies included questions regarding the experience of synaesthesia during the active period of the drug. Studerus (2013) reviews data from several studies (Studerus et al., 2010, 2011, 2012) that included nearly 600 psilocybin, MDMA, and ketamine test participants. Studerus (2013) provides prevalence rates of induced auditory-visual synaesthesia (only), with sounds as inducers, for these substances (MDMA: 10%; ketamine: 27%; psilocybin: 37%) and demonstrates its linear dose-dependent nature with psilocybin, the most studied substance in this context. Averaged across studies, the percentage of positive responses to questionnaire items relating to auditory-visual synaesthesia induced by psilocybin ranged from 0% for placebo and 45 mcg/kg doses, up to 50% for the highest psilocybin dose of 315 mcg/kg. The linear dose-synaesthesia relationship with psilocybin is also supported by independent data on synaesthesia as one of a number of visual effects in a similar indirect experiment (Griffiths et al., 2011) and tallies with earlier reports of less than 50% of participants experiencing experimentally-induced auditory-visual synaesthesia with 150 mcg/kg of psilocybin (Hartman and Hollister, 1963).

A notable finding of these studies is that psilocybin-induced auditory-visual synaesthesia is predicted by different demographic and individual difference variables, most notably *absorption*, the tendency to experience all-encompassing attentional states characterized by intense affective and imaginative involvement in an activity (Tellegen and Atkinson, 1974). Thus, individuals high in absorption appear to be more prone to drug-induced synaesthesia. Other studies administering psychedelics experimentally also report the prevalence of synaesthesia, usually defined as auditory-visual synaesthesia only. These studies have found prevalence rates ranging from 10 to 57% with various substances including ketamine, psilocybin, ayahuasca, *Salvia divinorum*, and *Ipomoea purpurea*, a plant containing LSD-like alkaloids (Savage et al., 1969; Lahti et al., 2001; Riba et al., 2004; Addy, 2010; Carhart-Harris et al., 2011).

Indirect experimental studies largely corroborate the results of the experimental studies. These studies possess a number of the same limitations, most notably the absence of behavioral measures to corroborate synaesthesia. In addition, they are exploratory in design and most studies only report on the prevalence of auditory-visual synaesthesias thereby obscuring our understanding of the types of synaesthesias induced by the chemical agents studied. However, some of the studies (Lahti et al., 2001; Riba et al., 2004; Addy, 2010; Carhart-Harris et al., 2011; Griffiths et al., 2011; Studerus, 2013) benefit from the inclusion of placebo controls, which in part circumvent confounds pertaining to demand characteristics.

### Case reports

We identified 17 case reports exploring the apparent induction of synaesthesia with chemical agents.

**Figure 1:**
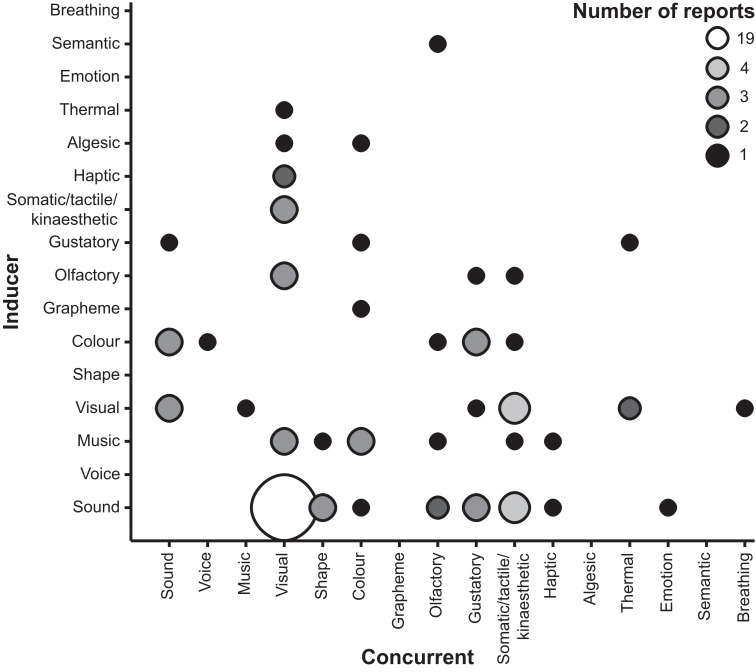
**Number of reports of particular inducer-concurrent associations in chemical-induced synaesthesias**. Smaller, darker markers reflect fewer reports.

The majority of the case studies simply report the form of synaesthesia that is induced, adding only to the known types that may be reported (see Table 1 and Figure 1), but a few studies provide further details regarding phenomenological characteristics of synaesthesia or information regarding chemical agents that can modulate congenital synaesthesia. One notable finding among case reports is Klüver (1966) observation of variability in the perceived visuospatial location of visual concurrents in induced auditory-color synaesthesia. Specifically, he reported that some participants would experience concurrents as endogenous mental images or representations whereas others would experience concurrents as though they were localized in space. This variability closely mirrors individual differences among synaesthetes. In particular, there is evidence that one subset of grapheme-color synaesthetes experience colors as mental representations (*associators*) whereas another experiences colors as spatially co-localized with the inducing grapheme (*projectors*) (Dixon et al., 2004; Ward et al., 2007). Klüver (1966) further noted that mescaline-induced synaesthesia can sometimes lead to quite complex fusions of several sensory percepts and may even produce associations between abstract concepts (e.g., negation) and visual images (e.g., a white square metal plate). One case report describes the apparent consistent induction of grapheme-color synaesthesia with melatonin, the veracity of which was supported with a behavioral measure of texture segregation (Brang and Ramachandran, 2008).

Of special interest are case reports describing the modulation of congenital synaesthesias with chemical agents. Brang and Ramachandran (2008) described the inhibition of an unspecified form of congenital synaesthesia with two types of antidepressant: the selective serotonin reuptake inhibitor, *fluoxetine*, and the substituted amphetamine, *bupropion*. The former result provides further support for the role of serotonin in the modulation of synaesthesia and thus complements direct and indirect experimental studies pointing to serotonergic agonists as reliable inducers of synaesthesia in non-synaesthetes.

As Shanon (2003) notes, case reports of chemically-induced synaesthesia are typically of the auditory-visual variety, particularly auditory-shape and auditory-color, as occurs with mescaline (Smythies, 1953; Klüver, 1966; Marks, 1975), LSD (Pahnke and Richards, 1966; McKenna, 1982; Hofmann, 1983), cannabis (Marks, 1975), and ayahuasca (Shanon, 2003; Fotiou, 2012). Such auditory imagery is sometimes reported to be dynamic in nature fluctuating with the sounds as they change (e.g., Pahnke and Richards, 1966; Hofmann, 1983), as when listening to music.

### Surveys

A small number of surveys report on the prevalence and type of chemically-induced synaesthesias. These reports are typically indirect, sampling substance users and reporting on synaesthesia as one of a number of phenomena occurring under the influence of different substances. In one study (DeGracia, 1995), psychedelic substances in general were reported to induce synaesthesia in 50% of users with 62 respondents reporting 23 different types of synaesthesia, the most common of which was auditory-visual (52% of those reporting synaesthesia), including music and voice inducers. Similar prevalence rates are found in a survey of cannabis users (56%) who predominantly reported music-color synaesthesia (Tart, 1975). Even surveys of users of obscure psychedelic substances like 4-HO-MET have reported the induction of synaesthesia, although prevalence rates were not reported (Kjellgren and Soussan, 2011). Notably, lifetime prevalence of chemical induction of synaesthesia is relatively higher than for those induced in individual experimental trials, as might be expected.

We found one study that surveyed synaesthetes regarding the impact of chemical agents on congenital synaesthesia (Cytowic and Eagleman, 2009). This study reports on the relative effects of commonplace drugs among 1279 verified grapheme-color synaesthetes. Alcohol and caffeine tended to enhance (9% both drugs) or reduce (3% caffeine, 6% alcohol) synaesthesia to relatively comparable degrees. Cytowic and Eagleman (2009) also report that of six synaesthetes ingesting LSD, two (33%) reported enhancing effects with the remainder reporting no effect. Although preliminary, these results suggest that LSD has stronger effects than commonplace psychoactive substances.

### Types of synaesthesias

All but a small number of the studies reporting induction of synaesthesia in non-synaesthetes specified the inducer-concurrent associations (see Table 1 and Figure 1). The most commonly reported type was overwhelmingly auditory-visual (19 reports; 23%). In turn, the most frequent inducer was auditory stimuli including music and voices (39 reports; 47%), and the most frequent concurrents were visual experiences (43 reports; 52%). However, these figures are somewhat inflated by the exclusive reporting of auditory-visual synaesthesia in the indirect experimental studies. Cumulatively, these results suggest that non-synaesthetes report similar forms of synaesthesia whilst under the influence of certain drugs as congenital synaesthetes, but also other inducer-concurrent associations that to our knowledge have not been reported in research studies of congenital synaesthesia, such as visual-thermal associations.

## Discussion

Here we reviewed the known studies describing the apparent induction and modulation of synaesthesia with chemical agents. These studies strongly suggest that chemical agents are capable of producing synaesthesia-like experiences. Crucially, there is large convergence across studies with different methodologies in terms of the prevalence of chemical-induced synaesthesia, the types of drugs (and neurochemical mechanisms) involved, and, to a lesser extent, the types of synaesthesias reported. There is also preliminary evidence that the same drugs have compatible effects on congenital synaesthesia. In what follows, we consider the limitations of these studies, the types of chemical agents implicated, and the types of synaesthesia reported. We conclude by considering whether these phenomena are comparable to congenital synaesthesia and offer suggestions for future research.

### Study limitations

Despite the importance of convergent results, this research literature suffers from a number of substantial limitations, which need to be considered when interpreting the veracity of the chemical induction of synaesthesia and any implications this research has for the neural mechanisms underlying congenital synaesthesia. First, there is a relative paucity of experimental studies. Of these, relatively few included placebo controls and some may be contaminated by demand characteristics. The absence of placebo controls in these studies is especially crucial because there is evidence that various psychedelic drugs enhance suggestibility (for a review, see Whalley and Brooks, 2009) and thus may augment participants’ susceptibility to demand characteristics. Furthermore, relatively little information is available regarding dosage, which may be crucial (Studerus, 2013), or the time course of the phenomenon (i.e., onset and duration). There is also considerable variability across participants with only a subset reporting chemical-induced synaesthesia. This variability may be explained in part by individual differences in absorption (Studerus, 2013), but has been largely ignored by researchers. The majority of the reviewed studies describe case reports and surveys. These types of studies are valuable at suggesting research avenues and possible mechanisms, but are not sufficiently rigorous to enable firm conclusions regarding the veracity of the effects.

The most severe limitation of the reviewed studies is that all but one (Brang and Ramachandran, 2008) relied on self-reports of experiences of synaesthesia. Although reports by congenital synaesthetes have been consistently validated by behavioral measures (Ward, 2013), it cannot be assumed that experiential reports among non-synaesthetes under the influence of chemical agents would translate to similar behavioral response patterns as congenital synaesthetes. Across studies, synaesthesia may not have been properly defined to participants or authors may have been using different definitions. Relatedly, some of the reported forms of synaesthesia [e.g., semantic-olfactory (Klüver, 1966); gustatory-semantic, (Breslaw, 1961)] closely resemble states of absorption (e.g., feeling cold when viewing a picture of an iceberg) (Ott, 2007). Given these limitations, caution must be exerted regarding the interpretation of the chemical induction of synaesthesia until these phenomena can be verified using more rigorous behavioral measures in studies with stronger experimental controls.

### Types of chemical agents

The studies reviewed here suggest that a wide range of drugs can produce synaesthesia-like experiences, even in controlled settings. Most of the research has included LSD, mescaline, psilocybin, ayahuasca, or MDMA. A notable commonality among these substances is that they are all serotonin agonists (e.g., Nichols, 2004), specifically serotonin 2A subtype agonists, although some non-serotonergic substances have been reported to induce synaesthesia, such as ketamine (Lahti et al., 2001) and *Salvia divinorum* (Addy, 2010). Few studies have included rigorous comparisons of these different drug classes, but the available evidence suggests that the prevalence rates for synaesthesia under the influence of serotonin agonists is greater than for drugs that do not target serotonin (Luke et al., 2012; Studerus, 2013). Furthermore, Simpson and McKellar (1955) reported that serotonin agonists both induced synaesthesia in non-synaesthetes and enhanced existing synaesthesias in congenital synaesthetes. This result was recently replicated in a survey of psychedelic users and congenital synaesthetes (Luke et al., 2012). Cytowic and Eagleman (2009) also reported the enhancement of congenital synaesthesia with LSD. Cumulatively, these results clearly implicate serotonin in synaesthesia and warrant further research on the role of this neurochemical in synaesthesia.

Nevertheless, other non-serotonergic compounds can also induce synaesthesia, although the most prevalent ones are typically also psychedelic in character, such as ketamine, *Salvia divinorum*, cannabis, nitrous oxide, datura, and dextromethorphan (Tart, 1975; Lahti et al., 2001; Addy, 2010; Luke et al., 2012; Studerus, 2013). These divergent chemical compounds act primarily on different neurochemical systems to each other and yet they all elicit profound changes in consciousness and somewhat similar phenomenological syndromes, such as the experience of synaesthesia. Given that there are currently around 350 known psychedelic chemicals and potentially 2000 untested ones (Luke, 2012), all with different modes of action, understanding exactly what neurochemical processes are responsible for the different experiential effects is a complex conundrum that remains to be disentangled by psychopharmacologists (Presti, 2011). Nevertheless, systemic taxonomical research that relates specific chemicals to particular experiences could illuminate the neurochemistry involved (Luke et al., 2012).

### Types of synaesthesia

There was consistent evidence across studies that auditory-visual synaesthesia was the most commonly experienced form of synaesthesia under the influence of drugs. In turn, auditory stimuli and visual experiences were the most common inducers and concurrents, respectively. However, there is also considerable variability across studies in the types of chemically-induced synaesthesias reported by participants. The source of this variability is at present unclear and may be driven by variability in the types of stimuli presented to participants and environment variability (most studies were not conducted in controlled laboratory environments). The preponderance of sound and music as inducers is likely due to the fact that participants commonly listen to music whilst consuming drugs. A number of studies also report only the prevalence of auditory-visual synaesthesias thereby inflating the differential prevalence rates between this type and other types of synaesthesia. Some phenomena labeled synaesthesia may actually be the result of other factors associated with the drug. For instance, we have found examples where drug-induced hallucinations (e.g., Cooles, 1980) that lack an unequivocal inducer-concurrent association pattern are incorrectly interpreted as synaesthesias (Ballesteros et al., 2006; see also Terhune and Cohen Kadosh, 2012). Finally, an increase in suggestibility following the ingestion of psychedelic drugs (Whalley and Brooks, 2009) may account for the occurrence of synaesthesia-like experiences that appear to be the product of absorption.

A common critique of this literature is that drug-induced synaesthesias tend to differ from congenital synaesthesias (Hubbard and Ramachandran, 2003; Sinke et al., 2012) in terms of the complexity and types of inducer-concurrent associations. The present review only partly supports this conclusion. On the one hand, there are reports of chemically-induced synaesthesias with unusual inducer-concurrent associations that, to our knowledge, have not been reported by congenital synaesthetes. One striking example is Klüver (1966) report of a complex visual-tactile-conceptual-visual-gustatory-olfactory synaesthesia following the ingestion of mescaline. Similarly, we do not observe any reports of particular well-documented types of synaesthesia such as spatial-sequence synaesthesia (Cohen Kadosh et al., 2012). On the other hand, there are examples of types of induced synaesthesia that are well-documented in the literature including grapheme-color (Brang and Ramachandran, 2008; Luke et al., 2012) and auditory-color synaesthesias (e.g., Hartman and Hollister, 1963). As an aside, we find it especially noteworthy that auditory-visual is the most frequently reported drug-induced synaesthesia as well as the most common acquired form of this condition (Afra et al., 2009). The under-representation of specific types of synaesthesia (e.g., grapheme-color, spatial-sequence) may reflect a lack of exposure to alphanumeric stimuli during drug consumption or, alternatively, may suggest that such types of synaesthesia require exposure to particular associations (e.g., Witthoft and Winawer, 2013) and/or take time to develop (e.g., Simner et al., 2009).

### Criteria of synaesthesia

An open question is whether chemically-induced synaesthesias are equivalent to congenital synaesthesias. Consensus has yet to emerge regarding the principal characteristics of synaesthesia and the ways by which ostensible synaesthesias can be confirmed as genuine. Nevertheless, there is considerable agreement that inducer-concurrent associations are automatic, consistent, specific, and accessible to consciousness (Ward and Mattingley, 2006; Deroy and Spence, 2013; Ward, 2013; Terhune et al., 2014). Here we use these criteria and other characteristics of congenital and chemically-induced synaesthesias to briefly consider the extent to which these two phenomena are similar.

Considered against these criteria, the available evidence indicates that chemically-induced synaesthesias do not as yet qualify as genuine synaesthesia. There is as yet no clear evidence that chemically-induced synaesthesias are automatic as all studies to date have relied on self-report except for one study (Brang and Ramachandran, 2008). The same goes for the criteria of consistency and specificity. There is one study that confirmed the consistency of melatonin-induced grapheme-color synaesthesia (Brang and Ramachandran, 2008), which is suggestive, but far from conclusive. The last criterion, accessibility to consciousness, however, appears to be overwhelmingly met by chemically-induced synaesthesias. These results clearly present a mixed picture but it should be noted that almost none of the studies have actually attempted to validate induced synaesthesias. Accordingly, any strong judgments regarding the veracity of chemically-induced synaesthesias in our opinion remain premature (for a different view, see Hochel and Milán, 2008).

Further evidence points to similarities and differences between congenital and chemically-induced synaesthesias. As previously noted, some researchers have emphasized that congenital synaesthesias tend to be relatively simple associations (Hubbard and Ramachandran, 2003; Sinke et al., 2012) whereas induced synaesthesias are often complex and sometimes reflect inducer-concurrent associations not observed in congenital synaesthesia. As observed in this review, there is considerable heterogeneity in the types of chemically-induced synaesthesias. As noted above, this variability may arise from different factors associated with the respective drug and environmental influences. Although the complexity and dynamism of psychedelic-induced synaesthesia is evident in some reports (e.g., Klüver, 1966) it is not mandatory (e.g., Simpson and McKellar, 1955), and indeed as Sinke et al. (2012) note, the complexity of the visual concurrent experience is related to dose and time from dosing (i.e., the intensity of the drug experience). It is notable that grapheme-color synaesthesia, the most well-studied form of congenital synaesthesia, was also reported by ~1% of recreational psychedelic tryptamine (e.g., LSD, psilocybin) users in an online survey (Luke et al., 2012). Furthermore, music and other auditory stimuli, the most common inducers across the studies reviewed here, function as inducers in more than 25% of cases of congenital synaesthesia (Hochel and Milán, 2008) (see above). Other notable similarities include individual differences in the visuospatial phenomenology of color concurrents in chemically-induced (Klüver, 1966) and congenital (Dixon et al., 2004; Ward et al., 2007) synaesthesias. Finally, the result that individuals high in absorption are more prone to chemically-induced synaesthesias (Studerus, 2013) is notable because absorption is indiscriminable from fantasy-proneness (Rhue and Lynn, 1989) and the fantasizing component of empathy is elevated among congenital synaesthetes (Banissy et al., 2013). Thus, individuals who are prone to chemically-induced synaesthesia may have a similar cognitive perceptual personality profile as congenital synaesthetes. To summarize, chemically-induced synaesthesias do not as yet meet accepted criteria for genuine synaesthesia, although no study has attempted to rigorously investigate this question. Induced synaesthesias do, however, display a number of striking similarities to congenital synaesthesias that warrant further attention.

### Future directions

The present review shows that there is convergent evidence that particular chemical agents produce synaesthesia-like experiences. However, the studies conducted to date suffer from numerous limitations and many questions remain unaddressed. Here we briefly outline further directions for research on the chemical induction of synaesthesia.

Future research on chemically-induced synaesthesia will need to utilize up-to-date methodologies to confirm that induced-synaesthesias are not the product of demand characteristics. In particular, there is a strong need for placebo-controlled, double-blind studies of these phenomena. There is consistent evidence that chemically-induced synaesthesias are more common with serotonin agonists, but experimental studies that directly compare a range of chemical agents are required before firm conclusions can be made. Future studies will also need to include established measures to verify the occurrence of synaesthesia such as measures of the automaticity (e.g., Dixon et al., 2004) and consistency (Eagleman et al., 2007; Rothen et al., 2013) of inducer-concurrent associations, rather than relying solely on self-reports as is the norm in the studies to date. Elsewhere, we have noted that some criteria (e.g., consistency) may not be applicable to genuine synaesthesia at an early stage because the associations may have yet to undergo consolidation (Terhune et al., 2014). Specifically, consistency of inducer-concurrent associations in congenital synaesthetes may arise through a consolidation process wherein the inducer and concurrent are repeatedly paired and the association is strengthened and becomes more specific over time. This hypothesis is consistent with research showing that inducer-concurrent consistency increases over time in children with synaesthesia (Simner et al., 2009). This should be considered when assessing the veracity of chemically-induced synaesthesias. Future studies would also benefit from the inclusion of more comprehensive phenomenological inventories in order to identify the similarities and differences between congenital and induced synaesthesias. Finally, it would be valuable to determine using transcranial magnetic stimulation whether drug-induced synaesthesias are dependent upon similar cortical structures (e.g., parietal cortex) as congenital synaesthesias (Esterman et al., 2006; Muggleton et al., 2007; Rothen et al., 2010). Pursuing these lines of investigation will help to elucidate whether chemically-induced synaesthesias are similar to congenital synaesthesias and thereby greatly inform further research on the neurophysiological and neurochemical mechanisms underlying congenital and chemically-induced synaesthesias.

## Summary and conclusions

Although it is nearly 170 years since the first report of the pharmacological induction of synaesthesia (Gautier, 1843), research on this topic remains in its infancy. There is consistent, and convergent, evidence that a variety of chemical agents, particularly serotonergic agonists, produce synaesthesia-like experiences, but the studies investigating this phenomenon suffer from numerous limitations. The wide array of suggestive findings to date are sufficiently compelling as to warrant future research regarding the characteristics and mechanisms of chemically-induced synaesthesias.

### Conflict of interest statement

The authors declare that the research was conducted in the absence of any commercial or financial relationships that could be construed as a potential conflict of interest.
